# Fellow-Workers and the Roll of Honour

**Published:** 1915-08-07

**Authors:** 


### ROUND THE WORLD.

[The Editor invites Early Information and Contributions Marked "Fellow-Workers."]

### Fellow-Workers and the Roll of Honour.

Captain Charles William Hope, R.A.M.C., who, it is understood, is going to serve on the Continent with a hospital sent out by the St. John Ambulance Association, is assistant surgeon to the Throat and Ear Department at King's College Hospital. His past appointments include those of house surgeon to the Royal Infirmary in Newcastle-on-Tyne, and assistant surgeon to the Golden Square Throat Hospital. He was for some time clinical assistant in the Throat and Ear Department of St. Mary's Hospital.

Amongst officers who have died from accident or natural causes since mobilisation must be included Lieutenant Andrew Philip, R.A.M.C., who had only just joined the Army. Qualifying at Edinburgh University in 1893, he became an M.D. Edin. a few years later, then becoming interested in public health work he took the D.P.H. of St. Andrews, and held the posts of medical officer of health for Burghs Alyth and Rattray, and school medical officer for Norfolk. Latterly he had been medical inspector of schools for the West Riding. Lieutenant Philip was in early years house surgeon to the London Temperance Hospital.

Referring further to the staff of the new hospital for officers at Euston (Endsleigh Palace Hotel), it may be noted that ophthalmology, neurology, and bacteriology are to be represented on its staff respectively by Mr. J. B. Lawford, Dr. J. Taylor, and Dr. G. T. Western.

Mr. John Bowring Lawford., who is an M.D. of McGill University as well as an F.R.C.S. Eng., occupies a leading position amongst British ophthalmologists. In this country he studied medicine at St. Thomas's Hospital, where he has since distinguished himself as ophthalmic surgeon. A well-known writer on his special subject, Mr. Lawford was at one time Editor of the *Ophthalmic Review*. He was president of the Ophthalmological Society of the United Kingdom 1911-13, and his present appointments include those of surgeon to the Royal London Ophthalmic Hospital and honorary ophthalmic surgeon to Spurgeon's Orphanage, Stockwell.

Dr. James Taylor, M.D. Edin., F.R.C.P. Lond., is physician to the National Hospital for Paralysis and Epilepsy in Queen Square, consulting physician to the Queen's Hospital for Children at Bethnal Green, and physician to the Royal London Ophthalmic Hospital. Dr. Taylor has written largely on neurological subjects, and co-edited an important work on "Diseases of the Nervous System" with the late Sir William Gowers.

Dr. George Trench Western, M.D. Camb., is senior assistant in the Inoculation Department of the London Hospital, and late pathological assistant there. He has carried out many important researches, particularly in connection with vaccine treatment and the bacterial reactions of the blood.

Dr. Graham Brown and Dr. Ninian Bruce, two leading Scottish neurologists, have been invited by the Lunacy Commissioners in charge of the special wards for mental shock and neurasthenia at the Royal Victoria Hospital, Edinburgh, to collaborate with them in the care of soldiers admitted thereto.

Dr. Hamilton Clelland Marr, the only one of these Commissioners not referred to in these notes last week, was a University of Glasgow student, taking the M.B., C.M. there in 1892, and subsequently adding the letters M.D. Glasg., F.R.F.P.S. Glasg. to his academic distions. A research scholar of Glasgow University, he was at one time medical superintendent of the Woodilee Mental Hospital, Lenzie, and before that senior assistant medical officer to the Crichton Royal Institution. A well-known writer on mental disease and idiocy, Dr. Carr is a past-secretary for Scotland of the Medico-psychological Association, and at one time lectured on physiology (as MacIntosh Lecturer) at St. Mungo's College, Glasgow.

Dr. Graham Brown enjoys a prominent place in the medical profession, being President of the Royal College of Physicians of Edinburgh. An M.D., C.M. Edin., F.R.C.P. Edin., he is physician to the Edinburgh Royal Infirmary and lecturer on neurology in the university. At one time Dr. Graham Brown, who is the author of a widely read manual on "Medical Diagnosis" and of a work on the "Treatment of Nervous Diseases," was formerly resident medical officer of the Chalmers Hospital, and an examiner in practical medicine for Edinburgh University.

Dr. Alexander Ninian Bruce is a brilliant young Edinburgh physician, who has had a remarkable academic career in the university of that city, in the course of which he won, amongst other awards, a gold medal at the M.D. Edin., the Dobbie Smith gold medal in botany, the Vans Dunlop scholarship in natural science, the Ellis prize in physiology, the Wightman prize in clinical medicine, the Patterson prize in clinical surgery, a Carnegie scholarship, the Goodsir memorial fellowship, and the Freeland Barbour fellowship of the R.C.P. Edin. Formerly assistant to the Professor of Physiology in the university, he now holds the posts of lecturer on physiological neurology there, and of senior clinical tutor and clinical assistant in the out-patient department of the Royal Infirmary. An F.R.S.E. Edin., in addition to being an M.D., Dr. Ninian Bruce is also a D.Sc. and M.R.C.P. Edin.

Dr. William Henry Willcox, the Home Office expert on poisons, who mobilised as captain in the 3rd London General Hospital (T.F.) on the outbreak of war, has been promoted to the rank of lieutenant-colonel and appointed consulting physician to the troops at the Dardanelles. One of the leading analysts in this country, Lieutenant-Colonel Willcox has appeared as the chief scientific witness at many famous criminal trials, the cause of justice having been furthered by his painstaking researches. An M.D., F.R.C.P. Lond., he is physician to out-patients and lecturer on public health and forensic medicine at St. Mary's Hospital, whilst formerly he was on the staff of the Great Northern Central.

Amongst other members of the St. Mary's Hospital staff serving in the Near East whose names have not yet been recorded in these notes must be included Captain D. C. L. Fitzwilliams, R.A.M.C., who is lecturer on clinical surgery and surgeon to out-patients at that institution. An M.D. Edin., Captain Fitzwilliams is also a Fellow of the Royal Colleges of Surgeons of both Edinburgh and England.

